# An empirical study on logistic service quality, customer satisfaction, and cross-border repurchase intention

**DOI:** 10.1016/j.heliyon.2024.e41156

**Published:** 2024-12-12

**Authors:** Guan Hui, Abdullah Al Mamun, Mohammad Nurul Hassan Reza, Wan Mohd Hirwani Wan Hussain

**Affiliations:** a UKM - Graduate School of Business, Universiti Kebangsaan Malaysia, 43600, UKM, Bangi, Selangor Darul Ehsan, Malaysia; b Business English, School of Foreign Languages, Changzhi University, Changzhi City, Shanxi Province, 046000, China; c Faculty of Business and Management, UCSI University, No. 1, Jalan Menara Gading, Cheras, 56000, Kuala Lumpur, Malaysia

**Keywords:** Logistics service quality, Cross-border e-commerce, Technology acceptance model, Customer satisfaction, Repurchase intention

## Abstract

Domestic e-retailers acknowledge logistics service quality (LSQ) as a critical success factor in business excellence. However, exponential growth in cross-border e-commerce (CBEC) requires a re-evaluation of the relationship between LSQ and consumers repurchase intention. By integrating the technology acceptance model, this study investigates the impact of LSQ on repurchase intention based on the LSQ (experience)–satisfaction–repurchase intention consequence chain. Data were collected from 466 Chinese consumers who had engaged in online shopping with international vendors. Partial least squares structural equation modeling was employed to examine the effectiveness of the research model. The results reveal that delivery service quality, return logistics services, and cross-border online shopping experiences significantly influence customer satisfaction. Nevertheless, the relationships between delivery information service, delivery stability, price fairness, and customer satisfaction are not supported. Price fairness, cross-border online shopping experience, and customer satisfaction positively affect cross-border repurchase intentions. Additionally, customer satisfaction mediates the relationships between LSQ, price fairness, cross-border online shopping experience, and repurchase intention. The novelty of this study lies in its focus on the cross-border dimension, introducing additional factors, such as price fairness and cross-border online shopping experiences, including other variables that affect LSQ and customer satisfaction, in contrast to domestic e-commerce. This study contributes to the literature by providing new insights into the complex dynamics of LSQ in CBEC and highlighting the nuanced role of customer satisfaction as a mediator in different market contexts. It offers empirical findings and valuable insights into the multiple dimensions of LSQ and customer satisfaction, thus contributing to the body of knowledge.

## 1 Introduction

Cross-border e-commerce (CBEC) has grown exponentially over the past few years. Data from iMedia Research indicate that CBEC imports in China reached 2.64 trillion yuan in 2019, a rapid increase of 17.3 % over the previous year. In 2020, analysts predicted that the cross-border import e-commerce market volume would reach 3.07 trillion yuan, and in 2021, they raised it to 3.55 trillion yuan. This growth rate is anticipated to accelerate further [1]. From a global perspective, e-commerce proliferation is expected to capture an increasing share of retail purchases. Projections indicate that online transactions will account for 20.1 % of retail purchases by 2024. Furthermore, the overall e-commerce market is anticipated to exceed $6.3 trillion in 2024 and is projected to reach $7.9 trillion by 2027 [2]. The projection for the quick commerce sector indicates a substantial expansion from $25 billion in 2021 to $72 billion by 2025, highlighting its accelerated growth and evolving consumer inclination towards more rapid delivery services [3]. Thus, to fulfil the demand for speed, there is a need for a transformation in logistics, where warehouses are evolving into dark stores that integrate all aspects of the delivery process [4]. Thus, logistics service quality (LSQ) is frequently cited as the most important factor in the competitive CBEC market for differentiating consumer experiences [5,6]. In the logistics industry, customer satisfaction is a critical determinant of repurchase intention. With the increasing globalization of trade and the rise of e-commerce, companies are now competing for service quality as much as price [7]. Understanding how LSQ influences customer satisfaction and, consequently, cross-border repurchase intention, is essential for developing strategies that enhance customer experiences and foster long-term loyalty. However, despite advancements in logistics technology and practice, challenges persist in maintaining consistent service quality across borders. Issues such as longer transit times, customs delays, and variability in service standards often lead to customer dissatisfaction, which, in turn, affects their willingness to make repeat purchases from international vendors. Therefore, improving the overall service quality of logistics remains an issue requiring further investigation. For instance, logistical service complaints received from Kaola.com include product loss and damage, improper order fulfillment, and delayed delivery. Products on Kaola.com are considered to have superior quality and trustworthiness, with stringent product quality control [8]. Nevertheless, some consumers do not prefer Sinotrans Limited, which is a contract logistics service provider. Hence, to meet the personalized logistics service requirements of clients, it is essential to increase the quality of overall logistics. Overall, the complexity of cross-border logistics requires a deeper understanding of how logistics service quality influences customer satisfaction and repurchase intention in a cross-border context [9]. Companies that excel in LSQ can differentiate themselves from their competitors and achieve significant operational advantages. Accordingly, understanding the relationship between LSQ, customer satisfaction, and repurchase intention enables organizations to focus on the most impactful aspects of their logistics services.

Previous research has examined the effect of the LSQ on customer satisfaction [[10], [11], [12]]. Some studies have also investigated the impact of LSQ on repurchase intention [13], eventually considering a local market [[14], [15], [16]]. Moreover, existing logistics studies have primarily focused on operational performance including cost reduction, time efficiency, and inventory management [17]. However, these studies have focused on customer satisfaction without fully exploring its effect on loyalty and repurchase behavior, especially in international e-commerce [18]. Thus, little research has been conducted on how LSQ relates to consumers’ satisfaction and impacts their intention to repurchase from foreign vendors in the international market [10]. Moreover, recent studies highlight the increasing complexity of cross-border logistics due to factors such as varying customs regulations, longer transit times, and cultural differences [19,20]. Nonetheless, a significant gap remains in understanding how these challenges impact LSQ and customer satisfaction in an international context. Additionally, existing literature indicates that the factors influencing customer satisfaction and repurchase intention may differ between consumer and industrial products, as well as between company sizes within the industrial sector [13]. Thus, empirical studies that delineate these differences in the context of CBEC are scarce. In addition, while some studies have identified a positive relationship between LSQ, customer satisfaction, and repurchase intention in specific sectors, such as food e-commerce [6], online retailing [21], and mobile shopping [22], there is a gap in quantitatively examining these relationships within the CBEC framework. Moreover, the role of customer satisfaction as a mediator between LSQ and repurchase intention in CBEC has not been quantified extensively. Oh, Kang [9] and Do, Kim [10] proposed developing and examining a comprehensive framework incorporating the multiple dimensions of LSQ and customer experiences to understand the underlying mechanisms that drive customer satisfaction and repurchase intention in CBEC.

In previous studies [23,24], the technology acceptance model (TAM) has been integrated into the logistics context. However, the current study introduces a more precise conceptual model [LSQ (experience)-satisfaction-repurchase intention consequence chain] to analyze the logistics service quality (i.e., delivery service quality, information delivery service, delivery stability, and return logistics service) that impacts customer satisfaction, eventually leading to repurchase intention, complementing TAM. This conceptual model emphasizes the differences in customer experience paths that lead to repurchase intention (cross-border online shopping experience) in the context of the LSQ. We also integrated cost performance (price fairness) [10,25] into our research model. As independent variables, these dimensions have rarely been examined in existing studies to measure their effect on customer satisfaction. Hence, to fill these research gaps, this study answers the following research questions.

(a) How do LSQs form an integrated model of satisfaction and repurchase intention within CBEC?
(b) Do cross-border online shopping experiences and price fairness influence consumer satisfaction and repurchase intentions in CBEC?

Theoretically, this study extends the LSQ to examine consumer satisfaction, which eventually leads to repurchase intentions. The proposed quality(experience)-attitude-intention chain integrated framework complements the TAM. By investigating the distinctive dynamics of LSQs and how they impact consumer satisfaction, leading to repurchase intention, this study, particularly within CBEC, makes a significant contribution to the body of knowledge. Moreover, this study delves into the direct impact of price fairness and cross-border online shopping experiences on customer satisfaction and repurchase intention to explore the interconnections between these constructs. In addition, this study elucidates the mediating role of customer satisfaction in the relationship between LSQ dimensions and repurchase intention, thereby providing a comprehensive understanding of this phenomenon. Thus, this study emphasizes the importance of a streamlined online shopping experience in fostering customer satisfaction and increasing repurchase intentions as a happy consumer is more likely to make a second purchase.

The remainder of this paper is organized as follows. Section 2 presents a comprehensive literature assessment and hypothesis formulation. The methodology is described in Section 3, and the results are presented in Section 4. Section 5 discusses the findings. Section 6 discusses the theoretical and practical implications, as well as the constraints and ideas for further study. Finally, Section 7 presents concluding remarks and highlights the achievements and major findings.

## 2 Literature review

We incorporated three phases in the literature review section. The initial phase portrays the main findings of the existing literature followed by the theoretical underpinnings of the service quality chain. Next, an inclusive discussion on the measurements of LSQ is presented considering the unique elements of CBEC. Finally, the LSQ and consumer satisfaction hypotheses are formulated within CBEC settings.

### 2.1 Key findings of the existing literature

LSQ is a critical determinant of customer satisfaction and repurchase intention, particularly in the context of CBEC. Do, Kim [10] highlighted the importance of LSQ in domestic e-commerce (DEC) platforms and suggested that CBEC moderates the relationship between LSQ, and consumer repurchase intention, enhancing the effect of personnel quality on satisfaction while attenuating the impact of timeliness and price fairness. This study provides valuable insights for DEC platforms expanding into foreign markets; however, further investigation is needed to refine the understanding of CBEC dynamics. Oh, Kang [9] supported the notion that LSQ positively influences customer satisfaction and intention to reuse, with delivery stability being a significant factor. It also identified eco-friendly logistics as a positive moderator between return logistics and satisfaction. While this study sheds light on the LSQ and its impact, additional variables (e.g., trust and price fairness) could enhance the model's explanatory power. Moreover, the generalizability may be limited to specific cross-border contexts or industries. Imannuel, Putri [26] studied the factors influencing Indonesian customers' purchasing intentions toward cross-border vendors in Indonesia's e-commerce platform. The results indicate that customer purchase intention is positively affected by price; however, reputation, perceived value, and trust negatively influence consumers' purchase intentions. This study focused on customer purchasing intentions without measuring customer satisfaction. Chen, Lan [27] conducted a systematic literature review of 40 CBEC studies. Although it provides valuable insights into consumer behavior, it may not directly address logistics services.

Akıl and Ungan [28] revealed positive impact of timeliness, order condition, order accuracy and order discrepancy influencing customer satisfaction, which in turn substantially affect customer loyalty in e-commerce logistics. While this study contributes valuable insights, it focused on a specific context (e-commerce customers in Turkey), therefore, the generalizability to other regions or industries may be limited. Further research could explore additional mediating factors beyond those studied here. Based on the semi-structured interview, Ali, Melkonyan [5] addressed sustainability practices in the logistics sector, particularly among logistics service providers (LSPs) in Egypt. It connects the sustainable LSQ framework with Egyptian law, emphasizing sustainability practices. However, the study did not demonstrate a connection between the factors. Baek, Lee [29] investigated how geographic cues embedded in a website affect Chinese consumers' cross-border shopping experiences. Participants in the “retailer's country” condition experienced higher telepresence and perceived greater product authenticity. The authors found that telepresence positively influenced trust in the retailer and perceived product authenticity, leading to favorable behavioral intentions. However, this study targets female Chinese consumers in their 20s and 30s who have bought Korean fashion products, which may not adequately represent all cross-border shoppers.

Gaudenzi, Confente [6] explored seven LSQ dimensions including reliability, security, timeliness, economy, pleasantness, convenience, and order accuracy and highlighted how different LSQ constructs can be combined to achieve high customer satisfaction through unique configurations. However, this study failed to demonstrate how customer satisfaction translates into repurchase intentions. Moreover, it focuses on Italian food companies, which may not fully represent other industries or geographical contexts. Gupta, Singh [11] studied service quality dimensions for LSPs in the Indian context. All five service quality constructs, including operational, resource, information, personnel contact, customization, and innovation, had a direct relationship with customer satisfaction, while these constructs indirectly influenced customer loyalty, with customer satisfaction acting as a mediator. Abbasi, Umer [30] conducted a study on the banking sector, which demonstrated that service quality has a substantial influence on customer satisfaction. This conclusion aligns with the findings in logistics research, suggesting that contented clients are more inclined to persist in utilizing logistics services and recommend them to others.

Overall, the literature repeatedly emphasizes the significant role of LSQ in enhancing customer satisfaction. However, few studies have specifically examined the impact of the LSQ on repurchase intention, apart from Do, Kim [10]. The current study shifts the focus towards exploring the varying influence of LSQ dimensions on customer satisfaction that leads to repurchase intentions, while concentrating on CBEC settings. This offers valuable insights for businesses aiming to refine their logistics strategies.

### 2.2 Theoretical foundation

The technology acceptance paradigm in behavioral science argues for a causal relationship between perception, attitude, intention, and behavior [31]. This model, which takes into account perceived benefits and risks, has also been used to understand online shopping behavior [32]. Additionally, the model's attitude is frequently interpreted as a summary of product or service evaluations [33]. Table 1 illustrates the customer satisfaction process, which is crucial to service quality chain models and coexists with the evaluation process [34,35]. Eventually, the logistics service industry implemented a service quality chain paradigm [13,36].

**Table 1:** Overview of the service quality chain model.

Theory	Literature	Context	Model Constructs					
			Quality	Experience	Value	Attitude	Intention	Behavior
Technology Acceptance Model	Venkatesh and Davis [31]	Information system adoption	External variables	Experience	Perceived usefulnes/ease of use	Attitude toward using	Intention to use	Actual system use
Value-Attitude-Intention Chain	Forsythe, Liu [32]	B2C sectors (Online shopping)			Perceived benefit/risk	Perception/current behavior	Intention to purchase	
Quality- Value-Attitude Chain	Boksberger and Melsen [34]; Gallarza, Gil-Saura [35]	B2C sectors (Online shopping)	Perceived Service Quality		Perceived Service Value	Customer Satisfaction		
Experience- Attitude-Intention Chain	Khalifa and Liu [37]; Wu and Chang [38]	B2C sectors (Online shopping)		Online shopping experience	Perceived usefulness	Customer satisfaction	Intention to purchase	
Quality-Attitude-Intention Chain	Miao, Jalees [36]; Jain, Gajjar [13]	B2C sectors (Online Shopping, Logistics)	Logistics service quality			Customer satisfaction	Intention to repurchase	
Quality (Experience) -Attitude-Intention Chain	Current study	B2C sectors (Cross-border online shopping, Logistics)	Logistics service quality, Price fairness	The cross-border online shopping experience		Customer satisfaction	Intention to repurchase	

However, in LSQ chain models, customers’ online shopping experiences have been measured using various methods [25,[37], [38], [39], [40]]: user-friendly interfaces, product information, visuals, personalization, payment security, shipping, and delivery options. Consequently, in CBEC, the online shopping experience should be measured comprehensively. Researchers have addressed the checkout process, effective communication, continuous feedback and improvement, and returns and exchanges, which appear vulnerable owing to higher delivery costs and longer delivery times [10]. Therefore, the growing importance of logistics in contemporary CBEC necessitates an inclusive study of the factors of LSQ to correlate customer satisfaction with repurchase intention [13,36].

Therefore, the current study proposes a new LSQ chain model for CBEC using two approaches. First, we contend that LSQ evaluation emphasizes cross-border e-retailers. This attitude corresponds to customer satisfaction because it denotes an evaluation procedure that conforms to the expectation-confirmation paradigm [41]. Second, the significance of price fairness in CBEC is no longer negligible because cross-border logistics are conducted via extended travel or airfreight to reduce the distance effect [42]. Therefore, this study integrated price fairness as a critical factor of LSQ impacting customer satisfaction and future purchases.

### 2.3 Logistics service quality

Perreault and Russ [43], the pioneers of LSQ research, argued that logistics operations create time, place, and utility for consumers. Therefore, the “seven Rs”—providing consumers with the appropriate quantity, of the perfect product, at the appropriate location and time, in the appropriate quality, at the appropriate price, and with the appropriate information [[44], [45], [46]]—increase product value. Mentzer, Gomes [47] recognized that in addition to physical distribution service aspects, consumers' perceptions of LSQ include additional consumer service elements, referred to as consumers' service marketing [44,48].

High-quality delivery services positively affect consumer satisfaction and a company's brand [12]. In the existing literature, logistics examined the costs of corporate activities [49]. As companies' technologies and management practices become more standardized, logistics services have been identified as competitive advantage strategies [45,50]. Accordingly, numerous studies across disciplines and nations have shown that LSQ improves both customer satisfaction and business performance, as well as a potential impact on customers' purchase intentions [44]. Personal contact, information, order quality, ordering processes, order discrepancy management, release quantities, order conditions, order correctness, and timeliness are the components that makeup LSQ, as described by Mentzer, Flint [51]. According to Gil Saura, Servera Francés [52], empathy and reliability determine the quality of logistics services. Bienstock, Royne [46] proposed logistics process quality (information, discrepancy, contact, and procedures) and logistics outcome quality (precision, availability, condition, and timeliness) in LSQ. A three-dimensional version of the LSQ scale was established by Gil-Saura, Servera-Francés [49]. This measure considers order quality, data quality, staff quality, timeliness, and order format.

Additionally, in defining service delivery quality, Rust and Oliver [53] emphasized identifying the components of service and improving these components to provide enhanced delivery. Researchers have categorized these components as technical (operational) and functional (relational) dimension quality [48,50]. In this study, we categorize order quality and timeliness as technical dimension and delivery service qualities and information quality as functional dimension quality. Owing to the significant distance and customs regulations of CBEC, undamaged and complete product delivery, managing returns, and refunds [21] could significantly improve customer experience. Therefore, this study adds return logistics services (technical dimension quality) and delivery stability (functional dimension quality) as independent variables to measure their effects on customer satisfaction. Cost performance (price fairness) was also added as a component of LSQ dissociated from the relational dimension of service performance [10,54].

### 2.4 Hypotheses development

#### 2.4.1 Delivery service quality

Delivery service quality determines the perception of satisfied customers about the on-time performance, reliability, and identification of their packages at every step of the process. It also refers to the condition of delivered goods [55]. According to the LSQ chain model, the quality of delivery services is a critical determinant of customer satisfaction. High delivery service quality meets or exceeds customer expectations, directly contributing to a positive overall experience and increasing satisfaction. From the customers’ point of view, a completed order that is aimed toward them does not affect the quality of the purchase [13,47]. This is because order requisitioning procedures are straightforward, order shipments are accurate, the received goods are in good condition, and problems are satisfactorily resolved [13]. Timeliness affects e-commerce customer happiness the most [56]. CBEC customer scenarios were studied by Oh, Kang [9], who examined whether variables such as speedy delivery and delivery at the desired time could impact customer emotions and attitudes. Accordingly, the result was significantly positive. Therefore, to validate further, we propose the following hypothesis.

H1 Delivery service quality positively influences customer satisfaction.

#### 2.4.2 Delivery information service

Delivery information services provide real-time package tracking and updates, which play a significant role in the LSQ model by providing transparency and reducing uncertainty during the delivery process. These services help manage customer expectations and increase satisfaction by keeping customers informed and reassured. Delivery information services allow senders and receivers to track their deliveries and obtain expected delivery timings [14]. High-quality information would likely benefit customers because of its assistance with product comparisons, informed purchasing, and increased transaction security [22]. Hong, Zheng [57] argued that delivery information services regularly update package statuses, including pickup, sorting, and delivery times. These upgrades enable users to track the packaging. Customers can also receive delivery status updates by e-mail, SMS, or mobile app push notifications when the package leaves the origin country, arrives in the destination country, or fails. This information positively influences customer satisfaction. Consequently, we propose the following hypothesis.

H2 Delivery information service positively influences customer satisfaction.

#### 2.4.3 Return logistics service

Return logistics services refer to the administration and procedures for processing customer product returns. It involves efficient and effective transportation, processing, and disposal of returned items [58,59]. The LSQ model emphasizes that an efficient and customer-friendly return process is essential for maintaining customer satisfaction. The policy outlines return conditions, including timeframes, permissible reasons, and associated fees or requirements. A clear return policy assists in managing consumer expectations and clarifies the return procedures [60]. Researchers have argued that it is essential to establish efficient processes for inspecting and evaluating returned products. The prompt and accurate evaluation of the conditions of returned products aids in determining the most suitable course of action, such as refurbishment, restocking, or disposal [61,62]. Automating and integrating return processing systems streamline return procedures [63,64]. Therefore, well-managed return services can improve customer happiness and the brand image. Thus, we propose the following hypothesis.

H3 Return logistics service positively influences customer satisfaction.

#### 2.4.4 Delivery stability

Delivery stability refers to the consistency and reliability of delivery services in terms of meeting delivery deadlines and providing customers with reliable and predictable experiences [9]. Stability in delivery services—consistency in meeting delivery timelines and quality—reinforces reliability, a key element in the LSQ model. Do, Kim [10] stated that delivery stability is characterized by the ability to consistently meet or deliver earlier than the promised delivery timelines. Generally, customers rely on estimated delivery dates [6].Thus, consistent adherence to these timelines can build customer trust and satisfaction [10]. Furthermore, the accurate delivery of correct items is an important aspect of delivery stability [65]. Ensuring accurate order picking, packing, and labeling helps prevent order errors and reduces the likelihood of incorrect or absent items being delivered [6,12].

Moreover, ensuring that the products are packaged appropriately to withstand transportation is essential for delivery stability. With sufficient packaging materials, protective measures and handling instructions may help prevent damage or breakage during transit [66]. Such initiatives can increase consumer satisfaction, strengthen business relationships, and promote global trade efficiency. Accordingly, we propose the following hypothesis.

H4 Delivery stability positively influences customer satisfaction.

#### 2.4.5 Price fairness

Price fairness is integrated into the LSQ model as a factor that influences customer perceptions of value. When a customer determines if a seller's pricing is reasonable or acceptable compared to a similar third party, it is referred to as price fairness [67]. Researchers have identified price policy as a critical aspect influencing customers' purchasing intentions [68]. Price policy also substantially impacts the relationship between an online retailer and its customers [69]. Consumers evaluate perceived prices based on their experiences or those of others. According to Rihn, Khachatryan [70], consumers have preconceived notions of reasonable price ranges for a given product. Therefore, purchase intent decreases if the price is perceived as unfair.

In the literature on logistics quality, price fairness refers to the perception or assessment of whether the price charged for logistics services, such as transportation, warehousing, or fulfillment, is reasonable and equitable [67]. Do, Kim [10] concluded that foreign vendors strive to establish and maintain price fairness, contributing to customer satisfaction, long-term partnerships, and a positive reputation in the industry. Accordingly, the following hypotheses are proposed.

H5 Price fairness positively influences customer satisfaction.

H6 Price fairness positively influences the intention to repurchase.

#### 2.4.6 Cross-border online shopping experience

The LSQ model suggests that the overall shopping experience, including ease of navigation, payment options, and customer support, is integral to customer satisfaction. A positive cross-border online shopping experience, which considers the unique challenges of international transactions, significantly boosts customer satisfaction by addressing potential pain points in the process. Extending the concept of the online shopping experience [25], the cross-border online shopping experience refers to activities occurring before, during, and after the purchase of goods or services from international online retailers or marketplaces [71]. This allows customers to access a wide range of products from different countries and enjoy the convenience of shopping for comfort in their homes [25,40]. Generally, customers can explore unique or hard-to-find items that may not be available locally; hence, online shopping can expand their purchasing options [29]. Further, cross-border online purchasing enables consumers to compare prices across countries and take advantage of potential cost savings or favorable currency exchange rates [72]. Moreover, customers can be motivated to repurchase from overseas vendors because of clear information on shipping options, delivery timeframes, tracking capabilities, and the availability of international shipping to their location [10]. Therefore, the following hypotheses are proposed.

H7 Cross-border online shopping experience positively influences customer satisfaction.

H8 Cross-border online shopping experience positively influences intention to repurchase.

#### 2.4.7 Customer satisfaction

Customer satisfaction is a way of assessing how well products and services fulfill customer expectations after purchasing. Online purchasing reflects a customer's overall satisfaction with the purchase experience and includes factors such as product quality, delivery, and customer service [73]. It is widely acknowledged that content customers are more likely to repurchase from the same retailer, recommend the brand to others, and intend to make more future purchases. Their positive experiences contribute to developing trust and loyalty, increasing the probability of future purchases [13,21]. Therefore, e-satisfaction emerges as a significant behavioral outcome in the context of international online purchasing. Thus, we propose the following hypotheses.

H9 Customer satisfaction is positively related to repurchase intention.

H10a-f Customer satisfaction mediates the effects of delivery service quality, delivery information service, return logistics service, delivery stability, price fairness, and the cross-border online shopping experience on repurchase intentions.

## 3 Research methodology

**Fig. 1:**
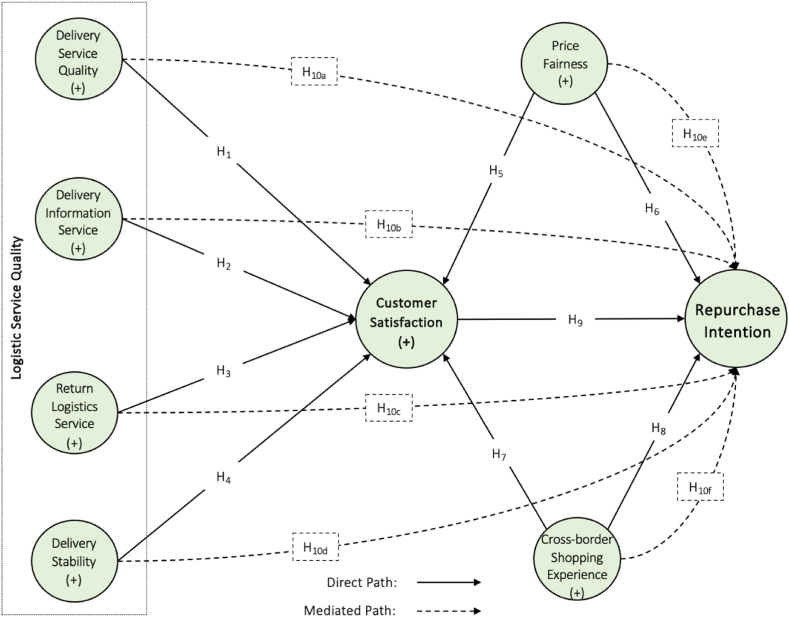
Research framework.

This study employed a deductive approach and quantitative method to explore the effects of LSQ, price fairness, and cross-border experience on repurchase intentions from foreign vendors. The framework is illustrated in Fig. 1. Unlike in longitudinal research, the data were gathered once from each respondent. We used the partial least squares structural equation modeling (PLS-SEM) to test the hypotheses.

### 3.1 Population and sampling

The Chinese consumers with direct purchase experience were the population of this study. A convenience sampling method was employed, targeting customers who had made at least one purchase from CBEC platforms in the past 6 months. This sampling technique was chosen for the current study due to its practicality and efficiency in accessing participants who had recent and relevant experiences with CBEC, allowing for timely and cost-effective data collection. This method is also suitable for exploratory research where initial insights are needed. The sample size was estimated using G-Power 3.1 software with eight predictors, a power of 0.95, and an effect size of 0.15. The smallest number of samples that needed to be analyzed with sufficient power was 160 [74]. Therefore, data retrieved from more than 160 respondents were considered statistically adequate for this study.

### 3.2 Survey instrument

The development of the survey instrument began with the generation of items for each construct, drawing on established scales from previous literature. To ensure relevance to CBEC, some items were adapted or modified to better reflect the specific nuances of this context. We employed straightforward and unbiased language in the questionnaire to ensure that all participants in this study could readily grasp the meaning of the instrument. To achieve this, we refrained from using complex terminology or complex sentence structures. Our goal was to make the survey accessible to respondents, regardless of their background or level of education. By using clear and concise language, we aimed to maximize the response rate and ensure that the data collected was accurate and reliable.

Six items were adopted [10,75] to capture delivery service quality. Moreover, Six items were adopted from Uvet [76] and Oh, Kang [9] to measure delivery information services. Referring to Oh, Kang [9], return logistics services were measured using six items. Furthermore, six items were retrieved from studies by Akıl and Ungan [28] and Oh, Kang [9] to assess delivery stability. Five items were adopted to measure price fairness [10,77], and six modified items were adopted to test the cross-border online shopping experience [29]. We also adopted five items from Park and Kim [78] and Zeithaml, Berry [79] to measure customers repurchase intentions from foreign vendors. Based on the previously explained concepts, a seven-point Likert scale ranging from 1 (strongly disagree) to 7 (strongly agree) was used for all questions. All items integrated in the study are included in the Supplementary Material S1.

### 3.3 Data collection

The survey was administered online, a method chosen for its efficiency and the ability to reach a broad audience. The questionnaire was developed in English and subsequently translated into Chinese by a professional translator. To mitigate potential cross-cultural discrepancies, three individuals who were proficient in both English and Chinese were recruited to conduct a pre-test. The feedback from these experts was incorporated to refine the wording of the questionnaire. Subsequently, a Chinese language specialist was engaged to verify the linguistic consistency of all items in the final version of the questionnaire. In addition to expert reviews, cognitive interviews were conducted with a small group of participants who matched the profile of the target population. These interviews helped identify any potential misunderstandings or ambiguities in the survey items, ensuring that the questions were interpreted as intended. Following these preliminary steps, a pilot test was conducted with a small sample of the target population. The issuance and completion of the questionnaires were closely supervised to ensure the validity and efficient collection of data. Thirty-seven questionnaires were distributed and collected during the pilot test. Subsequently, the pilot test findings confirmed the preliminary validity and reliability of the items used [80].

To ensure the accuracy and completeness of the responses, we selected 522 consumers who hailed from 31 administrative units that were directly subordinate to the Chinese central authority. These comprise 20 provinces (except Qinghai and Tibet, whose high-altitude and rugged terrain delay the development of logistics services), five autonomous regions, four municipalities (Chongqing, Beijing, Shanghai, and Tianjin), and two special administrative regions (Hong Kong and Macau) that makeup China's administrative divisions. Before e-mailing the questionnaires, the target consumers were called to identify the best representative who would consent to participate. An online survey form (Link: https://www.wjx.cn/vm/YmlbuDJ.aspx) was used to collect data from March to April 2023 from consumers with cross-border purchasing experiences. A few qualifying questions were added to the survey to confirm that data were collected only from the target respondents. Apart from this, ethical considerations were a priority throughout the data collection process. Consent was obtained from all participants, with assurances that their anonymity and the confidentiality of their responses would be maintained. This approach enabled us to minimize the likelihood of socially desirable responses. Thus, this study adhered to ethical guidelines, ensuring that participants were treated with respect and that their data was handled with care. Following all these steps, a total of 522 consumers were invited to participate in the survey. Consequently, this study received 466 valid responses, with a response rate of 89.3 %.

### 3.4 Common method bias

We carefully prepared measurement items to apply procedural remedies for common method bias (CMB). We also informed respondents that there were no correct or incorrect answers to the questionnaire and that their replies would be reviewed anonymously [81]. Harman's one-factor test was used to examine the impact of prevalent method bias on the current inquiry. According to that test, the contribution of the factor was 28.33 %, which is less than the 50 % cutoff for significance [82], confirming that CMB was not an issue for this study. Further, following the guidelines of Kock, Berbekova [83], this study assessed the total collinearity of all constructs to evaluate CMB. Table 2 displays the results for each construct using the shared variables and the corresponding variance inflation factors (VIF) values. All VIF values less than 3.3 indicate no bias in the single-source data.

**Table 2:** Full collinearity test.

	DQ	DI	DS	RL	PF	CSE	CS	RI
Variance Inflation Factors	1.415	1.388	1.438	1.392	1.469	1.369	1.366	2.105

**Note:** DQ: Delivery Service Quality, DI: Delivery Information Service, RL: Return Logistics Service, DS: Delivery Stability, CS: Customer Satisfaction, PF: Price Fairness, CSE: Cross-border Shopping Experience, RI: Repurchase Intention.

### 3.5 Multivariate normality

Using Web Power, we evaluated whether the multivariate data follows a normal distribution or not. Accordingly, the multivariate skewness, kurtosis, and *p*-values of Mardia were assessed. According to the results, multivariate skewness and kurtosis p-values for Mardia were found to be less than 0.05, indicating multivariate non-normality of the data.

### 3.6 Data analysis method

We used PLS-SEM method to analyze the relationships between latent variables. This is particularly useful when addressing complex models or small sample sizes and suitable for datasets with multivariate non-normality. Hair, Sarstedt [84] recommended using variance-based structural equation modeling to examine non-normal data. This study employed PLS-SEM in two phases. The reliability and validity of the model were assessed using an estimation procedure [85]. The associated evaluation is discussed below.

[84]. The path values (*β)* represent the impact of the input variables on the output variables [85]. Analysis using the coefficient of determination (*r*^*2*^), and effect magnitude (*f*^*2*^) can explain the change in the endogenous construct effected by exogenous constructs.

## 4 Findings

### 4.1 Demographic characteristics

As shown in Table 3, the aggregate ratio of female-to-male respondents is 64.6 %–35.4 %, indicating that significantly more women buy online than men. It was observed that 38 % of the participants were aged 18–25, 30.7 % were aged 26–35, 15.5 % were aged 36–45, 18.6 % were aged 46–55, 10.1 % were aged 56–65, and 1.3 % were aged >65 years. Moreover, 14.4 % of the 446 respondents had a diploma or advanced diploma, 62.4 % respondents had a bachelor's or equivalent degree, 11.8 % had a master's or equivalent degree, 4.4 % had a doctorate-level education, and 24.2 % had other qualifications.

**Table 3:** Demographic characteristics.

	N	%		N	%
*Gender*			*How long have you been shopping oversea*		
Male	165	35.4	less than one year	82	17.6
Female	301	64.6	1–3 years	94	20.2
Total	466	100.0	4–6 year	112	24.0
			7–9 years	52	11.2
*Age*			More than 10 years	126	27.0
18-25	177	38.0	Total	466	100.0
26-35	143	30.7			
36-45	72	15.5	*CBEC platform you used the most*		
46-55	47	10.1	Tmall International	115	24.7
56-65	21	4.5	JD Global Purchase	96	20.6
>65	6	1.3	Amazon Purchases Abroad	53	11.4
Total	466	100.0	Netease Kaola	36	7.7
			Vipin International	47	10.1
*Education*			Xiaohongshu	22	4.7
Diploma/Advanced Diploma	67	14.4	Suning Overseas	30	6.4
Bachelor Degree or Equivalent	291	62.4	Jumei Speed Duty-free Shop	12	2.6
Master or Doctorate Degree	101	21.7	Honey bud	28	6.0
Other	7	1.5	Gome	53	11.4
Total	466	100.0	Others	22	4.7
			Total	466	100.0
*Frequency of online shopping*					
Rarely (∼2 times)	7	1.5	*Favorite product to purchase*		
Occasionally (∼4 times)	7	1.5	Electronics and Media	28	6.0
Sometimes (∼6 times)	5	1.1	Furniture and Appliances	29	6.2
Frequently (∼8 times)	9	1.9	Food and Beverages	58	12.4
Usually (∼10 times)	66	14.2	Toys, Hobbies, and DIY	39	8.4
Always (More than 10 times)	372	79.8	Makeup	118	25.3
Total	466	100.0	Health & Medical	23	4.9
			Clothes	157	33.7
*Frequency of Cross-border online shopping*			Baby	14	3.0
Rarely (∼2 times)	11	2.4	Total	466	100.0
Occasionally (∼4 times)	7	1.5			
Sometimes (∼6 times)	44	9.4	*Average value of each purchase*		
Frequently (∼8 times)	53	11.4	1000 yuan ($145) or more	120	25.8
Usually (∼10 times)	7	1.5	501-1000 yuan ($73–145)	175	37.6
Always (More than 10 times)	342	73.8	301-500 yuan ($44–73)	120	25.8
Total	466	100.0	101-300 yuan ($15–44)	33	7.1
			Less than 100 yuan ($15)	18	3.9
			Total	466	100.0

**Note**: 1 USD = 6.35CNY.

When asked about their preferred online retailer, 115 (24.7 %) respondents selected Tmall International. JD Global Purchase was the second most-preferred online store during the pandemic, with 96 (20.6 %) respondents liking it. Meanwhile, Gome and Amazon Purchases Abroad ranked third, with 53 (11.6 %) respondents favoring it. Among them, 42.2 % selected other online merchants. The least preferred online purchasing sites were Vippin International (10.1 %), Netease Kaola (7.7 %), Suning Overseas (6.4 %), Honey Bud (6.0 %), Xiaohongshu (4.7 %), and the Jumei Speed Duty-free Shop (2.6 %).

Among the participants, 33.7 % favored online clothing shopping. Makeup (25.3 %) and food and beverages (12.4 %) were the next most popular products after clothing. Toys, hobbies, do-it-yourself, furniture and appliances, electronics and media, health and medical, and infant products followed, with smaller percentages.

A total of 79.8 % of the consumers preferred to shop online more than 10 times per year. A total of 16.1 % of the customers favored shopping online seven to ten times. Of all the customers, 4.1 % purchased online less than six times. Simultaneously, 73.8 % of customers preferred to purchase overseas online more than 10 times. Among the customers, 12.9 % preferred to shop online from international vendors seven to ten times, whereas 13.3 % preferred to shop online less than six times.

With a rate of 37.6 %, expenditures between 501 and 1000 yuan ($73 and $145, respectively) were the most common. The second most common spending ranges were 1000 yuan or more and 301–500 yuan or less, with a frequency of 25.8 % each. The least preferred spending ranges were 101–300 yuan ($15–$44) and less than 100 yuan ($15), with ratios of 7.1 % and 3.9 %, respectively.

### 4.2 Reliability and validity

Table 4 shows the validity and reliability of the constructs. The construct reliability was assessed using Cronbach's alpha, Dijkstra–Hensele's rho, and composite reliability (CR). Hair, Sarstedt [84] set the minimum Cronbach's alpha as 0.70. The results showed that each construct's alpha value was well above the 0.70 threshold, indicating adequate reliability of the constructs. Moreover, the lowest Dijkstra–Hensele rho value for the constructs was 0.874 [85], demonstrating sufficient reliability. According to Hair, Sarstedt [84], the suggested value of CR is higher than 0.70, and the results reveal that the values of all constructs meet the criterion, indicating the reliability of the constructs. Hair, Risher [85] also recommended establishing sufficient convergent validity for each construct to support the unidimensionality concept. To satisfy this criterion, the average variance extracted (AVE) must be greater than 0.50 for all items within each construct. In this study, all the items (Table 4) exhibit sufficient convergent validity. Finally, all the VIF values (Table 4) for each construct were significantly lower than the minimum value of 3.3, indicating that multicollinearity was not a concern for this study [84].

**Table 4:** Reliability and validity.

Variables	No. Items	Mean	Standard Deviation	Cronbach's Alpha	Dijkstra-Hensele's (rho_a)	Composite Reliability	Average Variance Extracted	Variance Inflation Factors
DQ	6	5.047	1.212	0.900	0.906	0.923	0.667	1.359
DI	6	5.471	1.006	0.887	0.893	0.914	0.640	1.230
DS	6	5.415	0.983	0.870	0.883	0.901	0.603	1.352
RL	6	5.297	1.014	0.879	0.885	0.908	0.623	1.332
PF	5	4.933	1.262	0.871	0.875	0.906	0.658	1.408
CSE	6	4.846	1.281	0.871	0.874	0.903	0.608	1.327
CS	6	5.196	1.042	0.909	0.909	0.929	0.687	1.146
RI	5	5.073	1.049	0.872	0.877	0.907	0.661	

**Note:** DQ: Delivery Service Quality, DI: Delivery Information Service, RL: Return Logistics Service, DS: Delivery Stability, CS: Customer Satisfaction, PF: Price Fairness, CSE: Cross-border Online Shopping Experience, RI: Repurchase Intention.

**Fig. 2:**
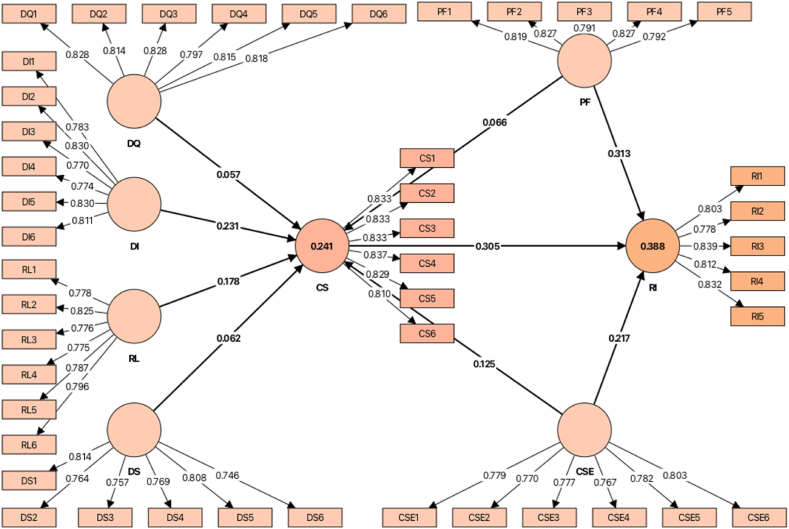
Measurement model with findings.

The Fornell-Larcker criterion and Heterotrait-Monotrait correlation ratio (HTMT) were applied to determine the discriminant validity of the constructs used in this study. According to the Fornell-Larcker criterion, the square root of each latent variable's AVE (diagonal values) should be greater than that of other items. Table 5 (Fornell & Larcker, 1981) shows that the Fornell-Larcker criterion was achieved. Furthermore, all HTMT ratios were <0.900 (Table 5), demonstrating that the latent variables had good discriminant validity [85]. As shown in Fig. 2, loadings attribute a given item to its corresponding latent variable [85]. All loading values were greater than the cross-loading values (please see Fig. 2 and Appendix 1). Thus, the findings indicated that all the items utilized in this study had adequate discriminant validity [86].

**Table 5:** Discriminant validity.

	DQ	DI	RL	DS	PF	CSE	CS	RI
*Fornell-Larcker criterion*								
DQ	0.817							
DI	0.296	0.8						
RL	0.369	0.319	0.777					
DS	0.371	0.262	0.366	0.79				
PF	0.372	0.251	0.396	0.331	0.811			
CSE	0.323	0.325	0.291	0.293	0.405	0.78		
CS	0.28	0.378	0.272	0.354	0.28	0.316	0.829	
RI	0.475	0.481	0.491	0.448	0.486	0.44	0.461	0.813
*Heterotrait-monotrait ratio (HTMT)*								
DQ								
DI	0.334							
RL	0.413	0.356						
DS	0.42	0.293	0.417					
PF	0.418	0.281	0.452	0.298				
CSE	0.363	0.366	0.331	0.567	0.462			
CS	0.305	0.417	0.298	0.298	0.311	0.353		
RI	0.535	0.546	0.567	0.567	0.553	0.501	0.511	

**Note:** DQ: Delivery Service Quality, DI: Delivery Information Service, RL: Return Logistics Service, DS: Delivery Stability, CS: Customer Satisfaction, PF: Price Fairness, CSE: Cross-border Online Shopping Experience, RI: Repurchase Intention.

### 4.3 Path analysis

**Fig. 3:**
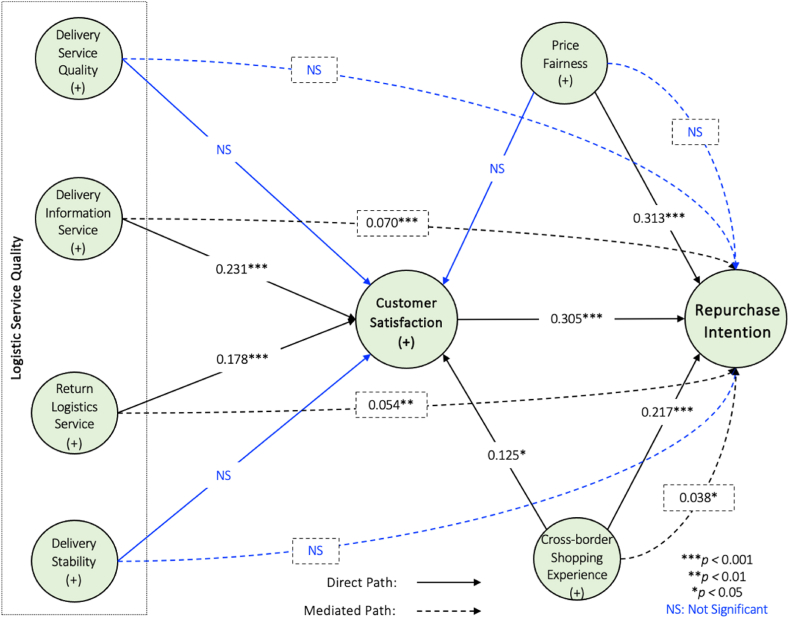
Final model with findings.

The empirical findings (Table 6 and Fig. 3) revealed that delivery information service (*β* value = 0.231, t-value = 4.638, *p* < 0.05), return logistics service (*β* value = 0.178, t-value = 4.638, *p* < 0.05), and cross-border online shopping experience (*β* value = 0.125, t-value = 2.331, *p* < 0.05) had significant positive effects on customer satisfaction; thus, supporting hypotheses H_2_, H_3_ and H_7_. Conversely, the influences of delivery service quality (*β* value = 0.057, t-value = 1.033, *p* > 0.05), delivery stability (*β* value = 0.062, t-value = 1.198, *p* > 0.005), and price fairness (*β* value = 0.066, t-value = 1.145, *p* > 0.005) were found to be insignificant for customer satisfaction; therefore, rejecting hypotheses H_1_, H_4_ and H_5_. The effect of price fairness (*β* value = 0.313, t-value = 6.219, *p* < 0.005), cross-border online shopping experience (*β* value = 0.217, t-value = 4.256, *p* < 0.005) and customer satisfaction (*β* value = 0.305, t-value = 7.303, *p* < 0.001) proved the significant repurchase intention of Chinese consumers; thus, accepting hypotheses H_6_, H_8_ and H_9_.

**Table 6:** Hypothesis testing.

Hypothesis		Beta	CI Min	CI Max	*t* values	*p* values	*f*^*2*^	*r*^*2*^	Decision
H_1_	DQ → CS	0.057	−0.032	0.152	1.033	0.151	0.003		Reject
H_2_	DI → CS	0.231	0.148	0.313	4.638	0.000	0.057		Accept
H_3_	RL → CS	0.178	0.094	0.260	3.552	0.000	0.031	0.241	Accept
H_4_	DS → CS	0.062	−0.018	0.153	1.198	0.116	0.004		Reject
H_5_	PF → CS	0.066	−0.029	0.162	1.145	0.126	0.004		Reject
H_7_	CSE → CS	0.125	0.035	0.213	2.331	0.010	0.016		Accept
H_6_	PF → RI	0.313	0.231	0.396	6.219	0.000	0.130		Accept
H_8_	CSE → RI	0.217	0.133	0.303	4.254	0.000	0.061	0.388	Accept
H_9_	CS → RI	0.305	0.234	0.372	7.303	0.000	0.132		Accept

**Note:** DQ: Delivery Service Quality, DI: Delivery Information Service, RL: Return Logistics Service, DS: Delivery Stability, CS: Customer Satisfaction, PF: Price Fairness, CSE: Cross-border Online Shopping Experience, RI: Repurchase Intention.

The structural model was evaluated by examining *r*^*2*^ and *f*^*2*^ using a consistent PLS approach [87]. The value of *r*^2^ reveals the entire amount of variation in an endogenous variable that may be attributed to exogenous variables comprising various factors. It is calculated by adding all the contributions made by the exogenous variables to the endogenous variable. According to the general guidelines suggested by Hair, Hult [86], *r*^*2*^ values of 0.75, 0.50, and 0.25 indicate substantial, moderate, and insignificant effects of exogenous variables on endogenous variables, respectively. Therefore, the results show small effects of the independent variables on customer satisfaction and moderate explanatory power on repurchase intention (Table 6). Combined with *r*^*2*^, *f*^*2*^ shows the individual effects of exogenous variables on endogenous variables by assessing changes in *f*^*2*^ when predictors are added or removed from the structural model [88]. Values of *f*^*2*^ greater than zero, 0.02, 0.15, and 0.35 indicate a small, average, and large effect of an independent variable on a dependent variable, respectively [89]. Based on this criterion, the results of this study showed a small effect for all variables.

### 4.4 Indirect effects

Table 7 shows that customer satisfaction did not mediate the association between delivery service quality and repurchase intention (*β* value = 0.018, t-value = 4.638, *p* > 0.05), delivery stability and repurchase intention (*β* value = 0.019, t-value = 1.143, *p* > 0.05), and price fairness and repurchase intention (*β* value = 0.019, t-value = 1.143, *p* > 0.005) relationships. However, a significant positive mediating effect of customer satisfaction exists between delivery information service and repurchase intention (*β* value = 0.070, t-value = 3.491, *p* < 0.05), return logistics service and repurchase intention (*β* value = 0.054, t-value = 3.028, *p* < 0.005), and cross-border online shopping experience and repurchase intention (*β* value = 0.038, t-value = 2.314, *p* < 0.05) relationships.

**Table 7:** Indirect effect.

Hypothesis	Association	Beta	CI Min	CI Max	*t* values	*p* values	Decision
H_10a_	DQ → CS → RI	0.018	−0.009	0.049	0.991	0.161	Reject
H_10b_	DI → CS → RI	0.070	0.040	0.106	3.491	0.000	Accept
H_10c_	RL → CS → RI	0.054	0.026	0.085	3.028	0.001	Accept
H_10d_	DS → CS → RI	0.019	−0.005	0.049	1.143	0.127	Reject
H_10e_	PF → CS → RI	0.020	−0.009	0.049	1.149	0.125	Reject
H_10f_	CES → CS → RI	0.038	0.011	0.065	2.314	0.010	Accept

**Note:** DQ: Delivery Service Quality, DI: Delivery Information Service, RL: Return Logistics Service, DS: Delivery Stability, CS: Customer Satisfaction, PF: Price Fairness, CSE: Cross-border Online Shopping Experience, RI: Repurchase Intention.

## 5 Discussion

This study classified LSQ into delivery service quality, delivery information service, delivery stability, and return logistics services and investigated their impact on customer satisfaction towards repurchase intention. Additionally, the effect of price fairness and cross-border online shopping experiences on customer satisfaction and repurchase intention were also examined. The following are some important findings of this study.

First, the results showed that delivery information services (H_2_), return logistics services (H_3_) and cross-border online shopping experiences (H_7_) had significant impact on customer satisfaction. Moreover, price fairness (H_6_), cross-border online shopping experiences (H_8_) and customer satisfaction (H_9_) significantly influenced repurchase intention. These findings indicate that customer trust is impacted by the speed, reliability, responsiveness, assurance, and flexibility of logistic services, which is consistent with the findings of Oh, Kang [9]. Further, these findings confirm that it is essential to simplify the return process for customers by providing prepaid return labels, coordinating with carriers to facilitate reverse logistics, and working with reliable shipping carriers or logistics partners to transport returned items promptly and securely. The conclusions of previous studies [56] highlighting the importance of delivery services and return logistics among CBEC logistics services are reaffirmed by this outcome. The significant impact of delivery information services and return logistics services on customer satisfaction suggests that transparency and convenience are critical to customer perceptions. In cultures where consumers value clear communication and hassle-free return processes, these findings are likely to generalize well. However, in regions where trust in online transactions is lower or where return processes are less standardized, the impact of these factors might differ. On the other hand, the significant impact of cross-border online shopping experiences on customer satisfaction highlights the importance of a smooth and reliable shopping journey. This finding is particularly relevant in culturally diverse markets, where expectations around customer service and logistical support can vary widely. In markets with high levels of cross-border shopping, such as the European Union, this relationship may be robust. However, in regions where CBEC is less common, additional factors such as payment security or customs processes might play a more significant role.

However, the relationships between delivery service quality (H_1_), delivery stability (H_4_) and customer satisfaction were insignificant. The results exhibit no statistical relationships between these variables, contradicting existing studies' findings [9,72]. This result may have occurred because of the vast distances involved and the unique conditions of cross-border transportation, both of which often result in lower expectations for delivery information and dependability. CBEC customers are already aware of the possibility of a decline in the quality of logistical information. They also perceive that the flow of their shopping experiences might be inconsistent. Moreover, Chinese consumers often prioritize speed and efficiency in logistics over other factors, potentially diminishing the relative impact of delivery service quality and stability on overall satisfaction. Additionally, China's e-commerce market is highly mature, with consumers accustomed to fast, reliable delivery services. In such a context, factors like delivery service quality and stability might not differentiate one service from another as significantly as in emerging markets. As a result, these variables might not strongly influence customer satisfaction, unlike in studies conducted in less mature markets where logistics performance varies more widely. These phenomena might produce a lower level of sensitivity in purchasing low-quality products. The insignificance of delivery service quality and stability on customer satisfaction might indicate that these factors are seen as baseline expectations rather than differentiators in some markets. In regions with highly reliable logistics infrastructure, customers may take these factors for granted, and their satisfaction may depend more on additional services, such as delivery information and returns. However, in less developed markets, where delivery stability and service quality are more variable, these factors might have a more pronounced impact on customer satisfaction. Moreover, the lack of a significant relationship between price fairness and customer satisfaction might reflect economic variability in consumer behavior. In markets where price sensitivity is lower, such as in more affluent economies, customers may prioritize service quality over price considerations. Conversely, in price-sensitive markets, price fairness can become a critical driver of satisfaction.

Contrary to previous research [10], the results of this study show that pricing fairness and customer satisfaction (H_5_) do not correlate. However, it affects repurchase intentions positively and significantly (H_6_), warranting further investigation. Customers are neither overly honest nor underappreciative because their logistics costs are high. This is mainly because consumers in China appear to place a higher value on intangible benefits, such as access to a broader selection or rarer goods that may not be readily available in their home markets. These can offset the elevated logistics expenses. Instead of focusing on price fairness, the highly competitive e-commerce market in China may lead to a reduced emphasis on customer satisfaction in terms of price fairness, as consumers may prioritize price over fairness. This has little impact on consumers’ desire to repurchase from foreign vendors. Further, it is important to note that the baseline model supports cross-border online shopping experiences. Positive online shopping experiences from foreign vendors, including seamless, secure, and convenient transactions, contribute to customer satisfaction and encourage repurchases in the global online marketplace.

Customer satisfaction was also confirmed with the mediation model between LSQ dimensions, price fairness, CBEC online shopping experience, and repurchase intention. Information delivery quality, return logistics services, and cross-border online shopping experiences include a full mediation effect. Conversely, delivery service quality, delivery stability, and price fairness exhibited insignificant mediating effects. This insignificant mediating effect may be attributed to certain consequences. For instance, in the highly competitive and mature Chinese e-commerce market, consumers may already expect high levels of delivery service quality and stability. This high baseline expectation could mean that these factors do not significantly enhance customer satisfaction when met, nor do they detract from it when slightly underperforming. As a result, any variation in these dimensions might have a limited impact on satisfaction, which in turn diminishes the mediating effect of satisfaction on repurchase intention. In addition, Chinese consumers often prioritize the speed and efficiency of delivery over other aspects of service quality and stability. If delivery is fast, minor issues in quality or stability may be overlooked, reducing their impact on overall satisfaction and, consequently, on repurchase intention. This focus on speed over quality may explain why customer satisfaction does not strongly mediate these relationships. Moreover, in the Chinese market, consumers are highly price sensitive, and competitive pricing is the norm. As such, perceived price fairness may not significantly affect satisfaction because consumers may view low prices as a standard expectation rather than as a differentiator. This cultural emphasis on competitive pricing could weaken the link between price fairness, customer satisfaction, and repurchase intentions. The findings suggest that in markets with less developed e-commerce infrastructure or where consumers are more price sensitive, these factors might have a stronger impact on customer satisfaction and, consequently, repurchase intention. To apply these findings to different jurisdictions, businesses should assess their local consumer expectations and economic conditions. In emerging markets or those with less reliable logistics services, enhancing delivery quality and stability can significantly boost customer satisfaction and loyalty. Similarly, in regions with higher price sensitivity, fair pricing strategies may play a more critical role in driving both satisfaction and repurchase intention. Tailoring strategies to meet the specific needs and expectations of each market will help businesses effectively enhance customer loyalty.

## 6 Implications

### 6.1 Theoretical implications

This study makes several theoretical contributions to the existing literature. First, we integrated the LSQ framework with TAM through a proposed quality (experience)-attitude-intention chain. This integrated framework provides a comprehensive understanding of how customers’ experiences with logistics services influence their attitudes and subsequent behavioral intentions, particularly in the context of CBEC. By combining these models, our study offers a more holistic view of the factors driving customer satisfaction and repurchase intentions, thus complementing and extending the TAM framework. Second, our study enriches the LSQ framework by exploring and validating specific dimensions—delivery service quality, delivery information service, return logistics service, and delivery stability—within the context of CBEC. These findings contribute to the literature by empirically validating the dimensions in a novel context. Third, existing literature primarily emphasizes the positive impact of LSQ on repurchase intention. However, our study provides a more nuanced understanding by examining both the direct and indirect effects of LSQ dimensions on repurchase intentions, particularly through the mediating role of customer satisfaction. This study contributes to the literature on virtual LSQ and offers insights into how these dynamics play out in digital platforms. Fourth, we tested the direct effects of price fairness and cross-border online shopping experience on customer satisfaction and repurchase intention. By incorporating these variables into the LSQ framework, our study adds a unique dimension to logistics services, highlighting the importance of perceived fairness and the shopping experience in shaping customer outcomes.

Fifth, this study extends the LSQ model to the context of CBEC, a sector with distinct logistical challenges. This extension offers new insights into how logistics service quality operates in international markets, an area that is underexplored in the literature. Sixth, our research helps clarify the inconsistencies in previous studies regarding the relationship between LSQ dimensions (such as delivery information services and delivery stability) [9,10], and customer satisfaction. Our findings suggest that these relationships may not be universally strong, particularly in the context of China's CBEC market, thereby contributing to a more refined understanding of these dynamics. Seventh, by conducting this study in the Chinese market, we provide insights into how cultural factors influence the application of the LSQ theories. This contextualization is essential for the global applicability of LSQ models and paves the way for comparative studies across cultural settings. Finally, the focus on virtual LSQ within digital platforms contributes to the evolving literature on logistics in the digital age, offering theoretical implications for how traditional logistics concepts apply in virtual environments, where customer experiences are mediated by technology.

### 6.2 Practical implications

The results of this study have important practical implications for businesses operating in CBEC markets. First, the significant impact of delivery information services on customer satisfaction suggests that companies should prioritize transparency and communication throughout the logistics process. By offering real-time tracking, regular updates, and accessible customer services, businesses can significantly enhance customer satisfaction. Logistics providers should provide customers with clear instructions on how to package and label returned merchandise, which includes providing return shipping labels or instructions for printing labels as well as packaging advice to prevent damage during shipment. In addition, the strong influence of return logistics services on customer satisfaction highlights the need for an efficient and customer-friendly return process. They should establish a simple and user-friendly return policy that specifies the circumstances, timelines, and procedures for returning merchandise and ensure an easy policy to find and publish prominently on the website. E-commerce companies should invest in simplifying return procedures, offering free returns, and ensuring that customers are informed of return policies. A robust return logistics service not only boosts customer satisfaction but also encourages repeat purchases as customers feel more secure in their buying decisions. Offering various return shipping options to accommodate customers’ varying preferences and requirements is also an effective strategy for international e-retailers. It provides customers with prepaid return shipping labels or allows them to choose their preferred shipping method. Therefore, global e-retailers should frequently evaluate their return logistics services to identify areas for improvement. They must analyze the reasons, trends, and customer feedback to resolve common issues and optimize the return procedure for increased customer satisfaction. Additionally, the significant positive effect of the cross-border online shopping experience on both customer satisfaction and repurchase intention underscores the importance of creating a seamless and enjoyable shopping experience for international customers. International vendors should provide prompt and effective customer service to answer questions, handle issues, and assist with cross-border shopping challenges. They should provide support channels, such as e-mail, live chat, or phone and ensure that customer inquiries are handled quickly and effectively.

However, the insignificant effect of delivery service quality, stability, and price fairness suggests that these factors may be viewed as basic expectations by consumers, particularly in mature markets, such as China. While it remains important for companies to meet these baseline expectations, they may not need to overinvest in areas beyond standard industry practices. Although customers might not feel more satisfied because of fair pricing, they are more likely to return if they perceive the pricing as fair. In the context of CBEC, e-commerce companies should emphasize clear communication about pricing, including currency conversion rates, taxes, and shipping costs to avoid any perception of unfair pricing.

Moreover, the valid mediating effect of customer satisfaction in the relationships between delivery information service, return logistics service, cross-border online shopping experience and repurchase intention implies that CBEC retailers should provide high levels of LSQ. For example, foreign vendors must accurately prepare customs declarations, classify goods, and adhere to the import and export regulations specific to each country. Moreover, they should collaborate with reputable carriers, shipping lines, airlines, or courier services to ensure the reliable and timely transport of goods across international borders. Furthermore, international e-retailers should provide customers with real-time updates on the status of their shipments, which helps manage customer expectations, improves transparency, offers multilingual customer support channels, and promptly responds to customer inquiries or concerns regarding cross-border shipments, enhancing customer satisfaction. In addition to identifying potential bottlenecks, developing contingency plans, and proactively managing the risks associated with product damage, transportation delays, and border issues, CBEC retailers must regularly review performance metrics, collect customer feedback, and implement enhancements based on these insights to ensure ongoing service excellence.

However, the insignificant mediating effect of customer satisfaction in the relationships between delivery service quality, delivery stability, price fairness, and repurchase intention suggests that although these factors may be important in other contexts, they do not directly influence customer satisfaction in a way that leads to increased repurchase intention in the Chinese market. For delivery service quality and stability, this could imply that customers in mature markets like China may see these as basic expectations rather than factors that significantly enhance their satisfaction or loyalty. While it is essential to maintain a certain standard of delivery quality and stability, businesses may need to explore other avenues to directly influence repurchase intentions. Moreover, the insignificant mediating effect of price fairness implies that, while fair pricing is crucial for encouraging repeat purchases, it may not be enough to boost customer satisfaction on its own. Therefore, businesses should ensure that their pricing strategies are transparent and competitive to foster repurchase intentions but should not rely on price fairness alone to drive customer satisfaction.

### 6.3 Limitations and future suggestions

The limitations of this study need to be addressed. First, while this study provides valuable insights into the examined relationships in the context of CBEC, it is important to note that the cross-sectional nature of the data limits our ability to draw causal inferences. Further research, particularly with longitudinal or experimental approaches, is necessary to confirm the observed relationships and to explore the potential causal mechanisms underlying these associations. Moreover, this study concentrates on an emerging country (China); therefore, generalization should be approached with caution. Future studies should be conducted on developing or other countries to gain comprehensive understanding. Further, cultural differences, such as uncertainty avoidance and individualism versus collectivism, are anticipated to influence the impact of LSQ on repurchase intention; thus, additional research is required in other geographical or cultural contexts. For example, in high uncertainty avoidance cultures, delivery stability might have a stronger influence on satisfaction. We also acknowledge that most of our respondents were between the ages of 18–35, which may limit the representativeness of our sample. Future research should include a more balanced age distribution to capture the perspectives of a wider range of consumers. Finally, further research could also explore how economic factors, such as consumer income levels and market competition, affect the relationship between LSQ dimensions, price fairness, and customer satisfaction.

## 7 Conclusion

This study investigated the impact of LSQ on customer satisfaction and cross-border repurchase intention, emphasizing delivery service quality, information delivery service, delivery stability, return logistics service, price fairness, and cross-border online shopping experience. We examined the relationships between these constructs and provided insights into consumer behavior in the context of CBEC by considering customer satisfaction as a mediator. The results demonstrate that delivery information service, return logistics service, and the cross-border online shopping experience significantly affect customer satisfaction. However, the delivery service quality and stability produced insignificant effect on customer satisfaction. The relationship between price fairness and customer satisfaction was also insignificant. Nonetheless, price fairness, cross-border online shopping experience and customer satisfaction positively influenced repurchase intention. The mediation analysis reveals that customer satisfaction significantly mediates the relationship between delivery information service, return logistics service, cross-border online shopping experience, and repurchase intention. However, the study also found that customer satisfaction does not significantly mediate the relationship between delivery service quality, delivery stability, price fairness, and repurchase intention. This study enhances the TAM by examining the influence of LSQ dimensions on the quality-attitude-intention chain, ultimately affecting repurchase intentions, in the context of CBEC. The findings of this study provide valuable insights into the complex dynamics of logistics service quality, customer satisfaction, and repurchase intention in CBEC, emphasizing the importance of specific logistics services and consumer experiences in driving customer loyalty in the competitive Chinese market.

## CRediT authorship contribution statement

**Guan Hui:** Writing – original draft, Methodology, Investigation, Conceptualization. **Abdullah Al Mamun:** Writing – review & editing, Methodology, Formal analysis, Conceptualization. **Mohammad Nurul Hassan Reza:** Writing – original draft, Methodology, Investigation, Conceptualization. **Wan Mohd Hirwani Wan Hussain:** Writing – review & editing, Methodology, Conceptualization.

## Ethical approval

The human research ethics committee of Changzhi University have approved this study (Approval Number: CZ-2023-0034). This study has been performed in accordance with the Declaration of Helsinki.

## Consent to Participate

Written informed consent was obtained from respondents who participated in the survey.

## Availability of data and materials

All data generated or analyzed during this study are included in this published article (Supplementary Material S2. Dataset).

## Funding

The study is supported via funding from (1) Department of Education of Shanxi Province (Grant no. 2021682) and (2) Shanxi Academy of Social Sciences (Grant no. YWQN202153).

## Declaration of competing interest

The authors declare that they have no known competing financial interests or personal relationships that could have appeared to influence the work reported in this paper.

## Figures and Tables

**Table:** Loading and cross-loadings

	DQ	DI	RL	DS	PF	CSE	CS	RI
DQ1	0.828	0.245	0.306	0.311	0.353	0.236	0.258	0.415
DQ2	0.814	0.248	0.325	0.314	0.283	0.292	0.200	0.363
DQ3	0.828	0.226	0.330	0.283	0.321	0.269	0.231	0.409
DQ4	0.797	0.267	0.332	0.349	0.315	0.289	0.186	0.400
DQ5	0.815	0.238	0.248	0.282	0.267	0.234	0.225	0.347
DQ6	0.818	0.236	0.276	0.292	0.281	0.277	0.252	0.388
DI1	0.234	0.783	0.254	0.215	0.207	0.274	0.279	0.414
DI2	0.223	0.830	0.233	0.186	0.203	0.237	0.319	0.377
DI3	0.256	0.770	0.248	0.193	0.240	0.226	0.270	0.357
DI4	0.258	0.774	0.223	0.207	0.132	0.237	0.263	0.396
DI5	0.279	0.830	0.319	0.273	0.250	0.297	0.348	0.419
DI6	0.179	0.811	0.247	0.178	0.166	0.284	0.322	0.351
RL1	0.273	0.219	0.814	0.335	0.320	0.204	0.250	0.369
RL2	0.279	0.238	0.764	0.281	0.319	0.242	0.208	0.432
RL3	0.273	0.176	0.757	0.244	0.315	0.219	0.165	0.378
RL4	0.272	0.176	0.769	0.266	0.263	0.214	0.183	0.354
RL5	0.336	0.220	0.808	0.254	0.331	0.273	0.255	0.371
RL6	0.292	0.176	0.746	0.327	0.293	0.198	0.177	0.399
DS1	0.310	0.273	0.310	0.778	0.264	0.225	0.297	0.345
DS2	0.326	0.289	0.286	0.825	0.238	0.235	0.329	0.386
DS3	0.274	0.235	0.256	0.776	0.255	0.257	0.238	0.359
DS4	0.250	0.253	0.269	0.775	0.243	0.235	0.249	0.334
DS5	0.280	0.193	0.283	0.787	0.281	0.225	0.261	0.364
DS6	0.294	0.258	0.324	0.796	0.292	0.217	0.285	0.332
PF1	0.339	0.227	0.311	0.303	0.819	0.359	0.214	0.421
PF2	0.270	0.203	0.282	0.352	0.827	0.333	0.252	0.425
PF3	0.281	0.147	0.217	0.314	0.791	0.319	0.177	0.343
PF4	0.330	0.240	0.266	0.343	0.827	0.331	0.275	0.399
PF5	0.286	0.191	0.258	0.293	0.792	0.297	0.206	0.373
CSE1	0.311	0.267	0.247	0.219	0.370	0.779	0.218	0.378
CSE2	0.216	0.214	0.189	0.176	0.306	0.770	0.211	0.300
CSE3	0.213	0.221	0.217	0.267	0.315	0.777	0.239	0.361
CSE4	0.172	0.266	0.226	0.229	0.269	0.767	0.243	0.307
CSE5	0.264	0.242	0.201	0.243	0.308	0.782	0.264	0.330
CSE6	0.318	0.305	0.279	0.224	0.320	0.803	0.295	0.372
CS1	0.235	0.322	0.276	0.200	0.233	0.243	0.833	0.390
CS2	0.247	0.298	0.265	0.247	0.238	0.287	0.833	0.353
CS3	0.238	0.319	0.300	0.163	0.264	0.252	0.833	0.378
CS4	0.241	0.342	0.279	0.281	0.211	0.252	0.837	0.413
CS5	0.238	0.286	0.303	0.225	0.203	0.275	0.829	0.373
CS6	0.193	0.312	0.337	0.234	0.242	0.266	0.810	0.382
RI1	0.345	0.391	0.340	0.410	0.381	0.353	0.368	0.803
RI2	0.410	0.352	0.355	0.368	0.362	0.334	0.275	0.778
RI3	0.417	0.425	0.365	0.392	0.401	0.377	0.464	0.839
RI4	0.360	0.353	0.360	0.420	0.387	0.354	0.357	0.812
RI5	0.398	0.427	0.401	0.406	0.440	0.368	0.388	0.832

**Note:** DQ: Delivery Service Quality, DI: Delivery Information Service, RL: Return Logistics Service, DS: Delivery Stability, CS: Customer Satisfaction, PF: Price Fairness, CSE: Cross-border Online Shopping Experience, RI: Repurchase Intention.
