# Supplementary material for: An empirical study on logistic service quality, customer satisfaction, and cross-border repurchase intention
Source: Heliyon. 2024 Dec 12;11(1):e41156. doi: 10.1016/j.heliyon.2024.e41156 (PMC11714389; doi:10.1016/j.heliyon.2024.e41156)
Supplement: Multimedia component 1 [file mmc1.doc]

**Supporting Material S1.** Survey Instrument

| Code | Items |
| --- | --- |
| DQ1 | 1. Most foreign vendors provide free/discounted shipping. |
| DQ2 | 2. Most foreign vendors complete delivery within the time frame |
| DQ3 | 3. Most foreign vendors provide a tracking system during shipping. |
| DQ4 | 4. Most foreign vendors provide flexibility to the rerouting package |
| DQ5 | 5. Most foreign vendors give me the flexibility to choose the delivery date |
| DQ6 | 6. Staffs of most foreign vendors are helpful, available, and responsive (during delivery). |
| DI1 | Most foreign vendors release information timely. |
| DI2 | Most foreign vendors release [precise](javascript:;) information. |
| DI3 | Most foreign vendors release [complete](javascript:;) information. |
| DI4 | Most foreign vendors offer to deliver a message to me |
| DI5 | Most foreign vendors address the delivery issue |
| DI6 | Most foreign vendors check delivery completion. |
| RL1 | The return policy of foreign vendors is listed on their website |
| RL2 | Most foreign vendors promise fast refunds and exchange |
| RL3 | Most foreign vendors take charge of the shipping fee for returning the commodities under any circumstances |
| RL4 | Most foreign vendors promise an easy return mode. |
| RL5 | Most foreign vendors provide return cargo tracking functions |
| RL6 | Most foreign vendors identify returns using wide criteria |
| DS1 | Most foreign vendors promise undamaged delivery |
| DS2 | Most foreign vendors promise delivery without lossless |
| DS3 | Most foreign vendors promise safe packing |
| DS4 | Most foreign vendors guarantee return and replacement in case of damage |
| DS5 | Most foreign vendors guarantee that the goods can be delivered to the precise destination on time. |
| DS6 | Most foreign vendors promise precautionary delivery |
| PF1 | Considering the benefits, the prices of products sold by most foreign vendors are fair. |
| PF2 | The shipping prices charged by most foreign vendors are reasonable |
| PF3 | In general, the prices charged by most foreign vendors seem reasonable given the cost of their manufacturing and marketing. |
| PF4 | Compared to the prices of other alternatives on the market, the prices of most foreign vendors are reasonable. |
| PF5 | Compared to the profit of other alternatives on the market, this foreign vendor’s profit is fair. |
| CSE1 | I have purchased high-quality products from foreign vendors. |
| CSE2 | I have purchased products from foreign vendors that could meet my needs exactly. |
| CSE3 | I have experienced helpful, responsive and punctual service which was provided by foreign vendors. |
| CSE4 | The products I purchased from foreign vendors were delivered to my home within seven days. |
| CSE5 | The delivery information of products I have purchased from foreign vendors was accurate, complete and timely. |
| CSE6 | Foreign vendors returned the products in their original price when there were flaws in the products. |
| CS1 | I was satisfied with the products quality received from most foreign vendors. |
| CS2 | I was satisfied with the customer service received from most foreign vendors |
| CS3 | I was satisfied with the pre-purchase experience from most foreign vendors (e.g., consumer education, product search, quality of information about the product, and product comparison). |
| CS4 | I was satisfied with the purchase experience from most foreign vendors (e.g, ordering, delivery date choice). |
| CS5 | I was satisfied with the post-purchase experience from most foreign vendors. (e.g, customer support, sales support, handling of returns/ refunds, delivery care). |
| CS6 | I was satisfied with the overall feeling of the online shopping experience from most foreign vendors. |
| RI1 | I will still give priority to foreign vendors if I have demands of buying the same products or services. |
| RI2 | I really enjoy shopping from foreign vendors. |
| RI3 | I will consume more from foreign vendors in the future. |
| RI4 | I will regularly purchase from foreign vendors in the future. |
| RI5 | I would like to introduce and recommend foreign vendors to others |

**Note:** DQ: Delivery Service Quality, DI: Delivery Information Service, RL: Return Logistics Service, DS: Delivery Stability, CS: Customer Satisfaction, PF: Price Fairness, CSE: Cross-border Online Shopping Experience, RI: Repurchase Intention
